# Atypical juxtaglomerular cell tumor in a young male with resistant hypertension and normal renin–aldosterone levels

**DOI:** 10.1016/j.eucr.2025.103280

**Published:** 2025-11-17

**Authors:** Mehdi Dadpour, Sara Besharati, Nima Saeedi, Mohammad Sajjad Zabihi, Emadoddin Hosseinjani, Mohammadreza Kamranmanesh, Reza Paeizi, Nooshin Ahmadi, Peyman Mohammadi Torbati

**Affiliations:** a Shahid Labbafinejad Medical Center, The Center of Excellence in Urology, Urology and Nephrology Research Center, Research Institute for Urology and Nephrology, Shahid Beheshti University of Medical Sciences, Tehran, Iran; b Department of Pathology, School of Medicine, Shahid Beheshti University of Medical Sciences, Tehran, Iran; c Department of Anesthesiology, Shahid Beheshti University of Medical Sciences, Tehran, Iran; d Department of Cardiology, Labbafinejad Medical Center, Shahid Beheshti University of Medical Sciences, Tehran, Iran; e Golestan University of Medical Sciences, Gorgan, Iran; f Research Institute for Endocrine Sciences, Shahid Beheshti University of Medical Sciences, Tehran, Iran; g Department of Pathology, Shahid Labbafinejad Medical Center, Shahid Beheshti University of Medical Sciences, Tehran, Iran

## Abstract

Juxtaglomerular cell tumor (JGCT), or reninoma, is a rare renin-secreting renal neoplasm that typically presents with severe hypertension, hypokalemia, and elevated renin and aldosterone levels. We describe an atypical case of a 21-year-old male with morbid obesity and resistant hypertension who exhibited normal biochemical findings despite the presence of a renal mass. He underwent partial nephrectomy, resulting in immediate normalization of blood pressure and subsequent regression of left ventricular hypertrophy. This case underscores the importance of considering JGCT in patients with refractory hypertension and a renal mass, even when the characteristic biochemical profile is absent.

## 1 Introduction

Juxtaglomerular cell tumor (JGCT), also known as reninoma, is a rare renin-secreting neoplasm of the kidney first described in 1967. Originating from the juxtaglomerular apparatus, it leads to autonomous renin production and secondary hyperaldosteronism, typically resulting in severe hypertension and hypokalemia.^1^ Clinically, JGCTs are categorized as typical functional, atypical functional, or non-functional types. The typical functional form is the most common and is characterized by elevated plasma renin activity, hyperaldosteronism, hypokalemia, and marked hypertension. The atypical functional type presents with hypertension without the complete biochemical triad, whereas the non-functional type lacks both hypertension and hormonal abnormalities.^2^^,^^3^

Although exceedingly rare, prompt recognition of JGCT is crucial, as surgical excision is curative and usually results in complete normalization of blood pressure and electrolyte levels.^4^ Here, we present a case of a 16-year-old male with a typical functional JGCT who presented with resistant hypertension, morbid obesity, and left ventricular hypertrophy (LVH). This case is notable for the coexistence of morbid obesity and severe, treatment-refractory hypertension requiring quadruple antihypertensive therapy at presentation.

## 2 Case report

A 16-year-old male with morbid obesity (BMI 41 kg/m^2^) was referred to our center for evaluation of a left renal mass incidentally detected on ultrasound study. He had an 8-years history of severe, refractory hypertension and some episodes of seizure and headache, with systolic blood pressure consistently ranging from 200 to 220 mmHg despite combination therapy with a calcium channel blocker, ACE inhibitor, beta-blocker, and diuretic. He had been hospitalized three times for hypertensive crises.

**Fig. 1:**
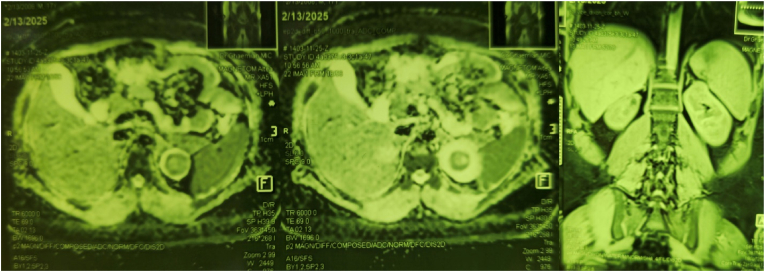
Contrast-enhanced MRI of the abdomen and pelvis demonstrated a 33 × 30 × 28 mm, well-circumscribed, partially exophytic mass located in the superior pole of the left kidney.

On patients's evaluation, cardiovascular findings suggested left ventricular strain, and echocardiography confirmed severe concentric left ventricular hypertrophy. Routine laboratory investigations revealed normal adrenal profile including plasma renin activity, aldosterone levels, serum potassium and 24-h urine metanephrine and nor-metanephrine. A metaiodobenzylguanidine (MIBG) scan was unremarkable, effectively ruling out pheochromocytoma to find any findings explain refractory hypertension. Contrast-enhanced MRI of the abdomen and pelvis demonstrated a 33 × 30 × 28 mm, well-circumscribed, partially exophytic mass located in the superior pole of the left kidney (Fig. 1). The lesion was isointense relative to the renal cortex on T1-weighted imaging, heterogeneously hyperintense on T2-weighted sequences, and showed restricted diffusion on DWI, consistent with a highly cellular lesion. A doppler ultrasound study performed to rule out renovascular hypertension but the results were completely normal.

**Fig. 2:**
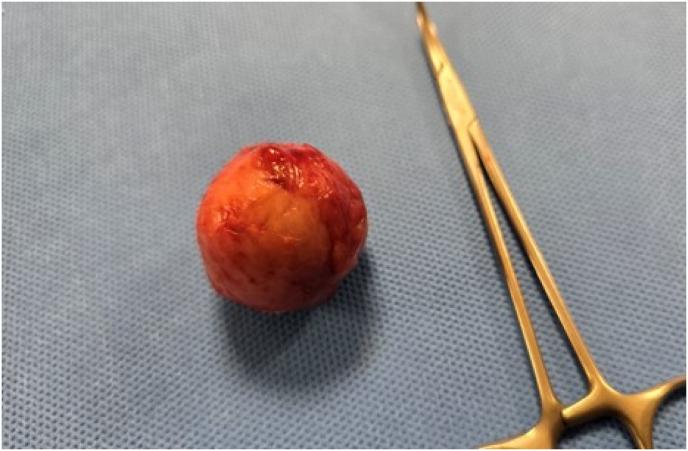
the specimen from partial nephrectomy of a 3 cm left renal upper pole mass.

A presumptive diagnosis of renal cell carcinoma led to surgical planning for partial nephrectomy^9^. Preoperatively, the patient's blood pressure remained poorly controlled despite maximal medical therapy including intravenous labetalol and nitroglycerin infusion for stabilization. He subsequently underwent partial nephrectomy under general anesthesia (Fig. 2).

Sectioning of 3 cm solid, greyish white mass revealed neoplastic tissue composed of sheets of polygonal to spindle cells with well-defined cell borders and central, round, uniform nuclei. A prominent vasculature component consisting of numerous small thin-walled and clusters of thick walled and hemangiopericytoma-like capillaries was also observed. Lymphoplasmacytic invasion was also present. Immunohistochemical (IHC) staining revealed a strong positive reaction for vimentin, CD34, and SMA, coupled with focal positivity for CD117. Negative reactions were reported for CD-31, -45, −99, CKAE1/AE3, EMA, S100, WT1, desmin, and HMB45. Ki-67 index was approximately 5 %. PAS staining was positive in the cytoplasmic granules of tumoral cells. The findings support the diagnosis of juxta-glomerular cell tumor.

Postoperatively, the patient's blood pressure normalized (110/70 mmHg) without the need for antihypertensive medication. He was discharged on postoperative day three. At three-month follow-up, he remained normotensive with no episodes of any headache and seizure, and repeat echocardiography demonstrated significant regression of left ventricular hypertrophy.

## 3 Discussion

JGCTs are uncommon renin-secreting tumors of the kidney, usually seen in young women presenting with difficult-to-control hypertension and hypokalemia.^5^ In contrast, our patient—a 16-year-old male with long-standing severe hypertension with normal potassium and plasma aldostrone—illustrates an atypical demographic and clinical evolution of this rare tumor.

In patients presenting with refractory hypertension and a renal mass, juxtaglomerular cell tumor (JGCT) should be included in the differential diagnosis.^6^ Typically, elevated plasma renin activity, secondary hyperaldosteronism, and hypokalemia are considered key diagnostic features and often guide clinicians toward the suspicion of a renin-secreting tumor.^7^ However, it is important to recognize that normal biochemical values do not exclude the diagnosis. Atypical cases, such as the present one, may exhibit normal renin, aldosterone, and potassium levels despite the presence of a functional tumor. Therefore, clinicians should maintain a high index of suspicion for JGCT in patients with resistant hypertension and a renal mass, even when the laboratory profile is inconclusive.^8^

Importantly, surgical excision of the lesion resulted in complete normalization of blood pressure and regression of left ventricular hypertrophy, underscoring the curative potential of early recognition and definitive management. This favorable postoperative outcome highlights the reversibility of secondary hypertension–induced cardiac remodeling once the source of excessive renin secretion is removed. Prompt diagnosis and surgical intervention are therefore essential not only for blood pressure control but also for preventing long-term cardiovascular complications associated with prolonged hypertension.

## 4 Conclusion

Juxtaglomerular cell tumor (reninoma) is an uncommon but treatable cause of secondary hypertension. While it usually presents with elevated renin and aldosterone levels and hypokalemia, normal laboratory findings do not rule out the diagnosis. JGCT should be suspected in patients with resistant hypertension and a renal mass, even without the classic biochemical features. Prompt recognition and surgical removal are essential, as curative resection can normalize blood pressure and reverse hypertension-related cardiac changes.

## CRediT authorship contribution statement

**Mehdi Dadpour:** Writing – review & editing, Writing – original draft, Supervision, Project administration, Methodology, Investigation, Conceptualization. **Sara Besharati:** Methodology, Investigation. **Nima Saeedi:** Methodology, Investigation. **Mohammad Sajjad Zabihi:** Writing – original draft, Data curation. **Emadoddin Hosseinjani:** Methodology, Investigation. **Mohammadreza Kamranmanesh:** Methodology, Investigation. **Reza Paeizi:** Methodology, Investigation. **Nooshin Ahmadi:** Investigation. **Peyman Mohammadi Torbati:** Validation, Supervision, Project administration, Investigation.
